# Rationale and process for N95 respirator sanitation and reuse in the coronavirus disease 2019 (COVID-19) pandemic

**DOI:** 10.1017/ice.2021.37

**Published:** 2021-02-02

**Authors:** Gregory J. Golladay, Kevin A. Leslie, Wilhelm A. Zuelzer, Anthony D. Cassano, Joshua J. Plauny, Frank E. Daniels, Gonzalo Bearman, Stephen L. Kates

**Affiliations:** 1 Department of Orthopaedic Surgery, Virginia Commonwealth University, Richmond, Virginia; 2 VCU Ventures, Virginia Commonwealth University, Richmond, Virginia; 3 Division of Thoracic Surgery, Department of Surgery, Virginia Commonwealth University, Richmond, Virginia; 4 Supply Chain, VCU Health, Richmond, Virginia; 5 High-Level Disinfection Unit, VCU Health System, Richmond, Virginia; 6 Division of Infectious Disease, Department of Internal Medicine, Virginia Commonwealth University, Richmond, Virginia

## Abstract

**Objective:**

The novel severe acute respiratory coronavirus virus 2 (SARS-CoV-2) was first reported in Wuhan, China, in December 2019 and is notable for being highly contagious and potentially lethal; and SARS-CoV-2 is mainly spread by droplet transmission. The US healthcare system’s response to the COVID-19 pandemic has been challenged by a shortage of personal protective equipment (PPE), especially N95 respirators. Restricted use, reuse, and sanitation of PPE have been widely adopted to provide protection for frontline healthcare workers caring for often critically ill and highly contagious patients. Here, we describe our validated process for N95 respirator sanitation.

**Design:**

Process development, validation, and implementation.

**Setting:**

Level 1, urban, academic, medical center.

**Methods:**

A multidisciplinary team developed a novel evidence-based process for N95 respirator reprocessing and sanitation using ultraviolet (UV) light. Dose measurement, structural integrity, moisture content, particle filtration, fit testing, and environmental testing were performed for both quality control and validation of the process.

**Results:**

The process achieved UV light dosing for sanitation while maintaining the functional and structural integrity of the N95 respirators, with a daily potential throughput capacity of ∼12,000 masks. This process has supported our health system to provide respiratory PPE to all frontline team members.

**Conclusions:**

This novel method of N95 respirator sanitation can safely enable reuse of the N95 respirators essential for healthcare workers caring for patients with COVID-19. Our high-throughput process can extend local supplies of this critical PPE until the national supply is replenished.

The novel coronavirus disease 2019 (COVID-19) was first reported in Wuhan, China in December 2019, and it is notable for being both highly contagious and potentially lethal.^1^ Severe acute respiratory coronavirus virus 2 (SARS-CoV-2) is mainly spread by aerosolized droplet and contact transmission. Patients infected with the virus exhibit a broad range of presentations, including asymptomatic infection, which has facilitated its rapid transmission.^2–4^ Patients with underlying comorbidities such as pulmonary disease or immunosuppression, and the elderly, are at particular risk of severe illness associated with respiratory failure requiring mechanical ventilation and a high rate of mortality.^4^ Testing capabilities have also been limited, and the only proven treatment to date is supportive care including mechanical ventilation.^5^ Increased testing capacity could allow for greater case identification, patient isolation, and contact tracing. Social determinants, such as crowded living conditions and race, appear to be associated with a higher prevalence of infection.^6^ Social distancing and stay-at-home orders, the closure of nonessential businesses, and the use of face masks have been widely instituted to help “flatten the curve” of new cases.^6^

SARS-CoV-2 is primarily transmitted by aerosolized droplets; thus, high-filtration face respirators and face shields provide an important means of protecting healthcare personnel from becoming exposed and infected.^7–10^ When properly fitted and worn, N95 respirators provide better protection than surgical/droplet respirators from inhaled aerosols and particulates.^9,11^ This protection derives from 2 primary factors: a tight fit to the wearer’s face and the engineered filter fabric capable of capturing airborne particles, dust, and mists. The certification of the respirators, N95 versus N99, represents the efficiency of the filter material (respectively 95% vs 99.7% removal of 0.3-µm particles) under test conditions but actual performance of the respirator during work tasks is highly dependent on the fit to the user. Employees at our institution undergo required annual fit testing to determine their appropriate respirator size and style. According to Occupational Safety and Health Administration (OSHA) regulation 1910.134, qualitative fit testing is conducted using a standard protocol that involves testing the ability of a person wearing the respirator to detect the odor of aerosolized saccharin or Bitrex (denatonium benzoate, Bitrex, Edinburgh, Scotland, UK). At our institution, a good fit is identified by the absence of the ability to smell saccharin while the test subject wears a plastic hood.

The large number of patients affected by COVID-19 globally induced a high demand for personal protective equipment (PPE), intensive care beds, ventilators, and disinfection supplies.^7^ In particular, N95 respirators, an essential PPE item, have been in particularly limited supply.^7,8,12^ As a result of N95 respirator supply shortage, regions treating a high number of COVID-19 cases early in the pandemic had to resort to subsatisfactory methods of protection of healthcare workers, including the use of self-made cloth masks or bandannas covering the mouth and nose to minimize exposure.^13,14^ Until the global supply chain and factory increase production to meet the demand for PPE, reuse and sanitation of N95 respirators could represent an alternative solution, provided that safety and efficacy of the device is maintained.^15^ UV sanitation has been shown to be effective in eradicating a wide range of pathogens including *Clostridium difficile*, *Mycobacterium tuberculosis*, bacterial spores, and viruses (including coronaviruses).^16^ A UV-C dose of 2–7 mJ/cm^2^ is sufficient for killing single-stranded RNA viruses (eg, SARS-CoV, MERS-CoV) on 2-dimensional nonporous surfaces.^15^ Concerns have been raised that UV sanitation of N95 respirators may result in reduced efficacy of the filter material and/or degradation of the straps or respirator structure.^17,18^ Any failure mode may result in reduced protection for the user of the respirator. High-dose UV irradiation has been shown to degrade polymers.^17,18^ Most N95 respirators are formed from woven polyester. Two commonly used respirators, the 3M 1860 respirators (3M, St Paul, MN) and Halyard Fluidshield respirators (Owens and Minor, Halyard, Alpharetta, GA) have 3 layers and 4 layers, respectively. Each particular layer will likely exhibit a different degradation pattern in response to repeated high-dose UV-C. The UV light exposure dose received by the outer (patient-facing) and innermost (healthcare worker-facing) layers will be greater than the middle layer(s) because of attenuation and shadowing. The aim of this manuscript is to describe our method of decontaminating N95 respirators for reuse with ultraviolet germicidal irradiation (UVGI) and to detail the effects of this process on respirator integrity and functionality.

## Methods

Our urban academic tertiary-care referral center has 811 beds, including 120 ICU beds. We have 2 vendors for N95 respirators, 3M 1860 and Halyard Fluidshield, and each vendor has a regular and small size. All protocols were developed to safely sterilize all 4 respirator types. Respirator users are instructed to avoid the use of face makeup to avoid soiling their respirator. All workers are instructed to label N95 respirators on the strap with their name and employee ID, using permanent marker, and surgical tape.

Following CDC guidance, respirators are discarded when contaminated with blood, respiratory or nasal secretions, or other bodily fluids from patients; when the respirator is damaged, deformed, or hard to breathe through; or when the straps are damaged or stretched and no longer taut enough to adequately hold the respirator to the face.^12^ Respirators that have been used but are structurally intact are collected in “dirty respirator” bins placed on the units treating COVID-19 patients and patients under diagnostic investigation for coronavirus infection, the operating room, and the emergency department. Subsequently, we have expanded the process to include all inpatient units.

We assessed several proposed methods of respirator sanitation, including low-temperature gas plasma sterilization (Sterrad, Advanced Sterilization Products, Irvine, CA), steam heat, ethanol, and UV light. We chose to use ultraviolet (UV) light because this method is both scalable and our hospital owns and has been able to rapidly deploy six mobile UV sanitation devices (Tru-D, Tru-D SmartUVC, Memphis, TN) that were being used to disinfect patient rooms and to terminally disinfect operating rooms.^16^ This device uses a circular array of Philips Healthcare (Amsterdam, Netherlands) low-pressure UVC lamps with a peak wavelength of 253.7 nm.

An unoccupied building on campus has been repurposed into a sanitation facility. A room (8.3 m × 4.25 m) was chosen for the sanitation process and was painted with reflective paint (SmartFINISH, Tru-D Smart UVC) to reduce total cycle time and maximize exposure on both sides of the respirators. Mapping of UV-C irradiance was validated at a range of distances, heights, and angles within the room. AG&R Labs (Santa Clara, CA) Model 220 NIST-traceable UVC meter was used to measure the irradiance. The meter’s probe was placed in the position and orientation of a respirator on 1 of the 6 trellises. The Tru-D Smart UVC was activated and allowed to run for 2 minutes to ensure a stable irradiance reading. This process was repeated twice for every respirator location on the trellis. In addition, measurements were taken in this fashion for the corners and centers of all other trellises and were in agreement. Because the Tru-D Smart UVC emits UV light uniformly in 360°, and because the trellises are of identical dimensions and are placed in a marked symmetrical orientation around the Tru-D, the irradiance patterns for all trellises should be uniform.

**Fig. 1.**
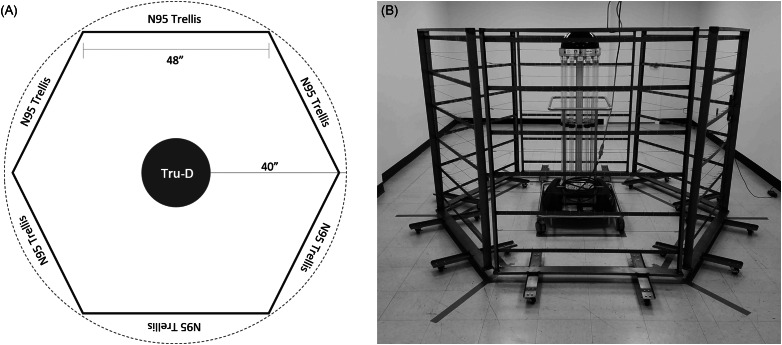
Room layout and trellis design.

We designed and constructed 2 models of trellis racks; one is narrow and tall and the other is short and wide; each is capable of holding 40 N95 respirators. The masks are spaced such that there is no overlap or shadowing. Trellises are then arranged symmetrically around the Tru-D Smart UVC with all patient-facing sides of the masks facing the device, in a hexagonal arrangement determined to be ideal for both workflow efficiency and appropriate UV-C dosing (Fig. 1).

**Fig. 2.**
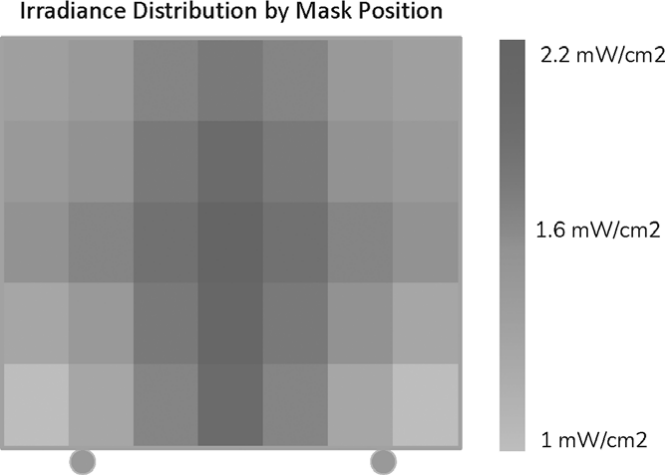
Mask irradiance by trellis location. Note. Ultraviolet dose = irradiance × seconds of exposure; mW, milliwatt; range, 1–2.2 mW/cm^2^.

Based on the irradiance pattern measured via our NIST-traceable meter, we found that the UVC light was distributed unevenly across the mask. We identified the least irradiated mask position on the trellis then measured the cumulative UVC dose received in that position. In addition, UV-C irradiance mapping revealed that the backside of the respirators was receiving significantly less light due to insufficient reflectivity caused by scatter and absorption, and that the location of a respirator relative to the UV source affected UV dosing (Fig. 2).

It takes 12 minutes to deliver 1,000 mJ/cm^2^ to this least irradiated position. Therefore, we used two 12.5-minute cycles deliver a minimum of 1,000 mJ/cm^2^ to each side of all masks. After the first dosing cycle, the trellises are turned 180° so that the wearer-facing sides of the masks are oriented toward the device. The range of doses received by each side of the masks on a trellis is 1,000–2,200 mJ/cm^2^.

To ensure accurate dosing and regularly assess quality control, a custom-built sensor system was deployed that measures, displays, and logs the real-time accumulated UV dose delivered to every batch of respirators.

When handling the respirators, all sanitation-facility personnel follow strict hygiene measures including handwashing and the use of full PPE (shoe covers, hats, impermeable gowns, gloves, eyewear, face shields, and N95 respirators). Dirty respirators are first inspected for structural defects.^12^ Damaged respirators are discarded and replaced with a laminated card listing the healthcare worker’s name and employee identification number, and after the sanitation cycle they are replaced with a new, labeled respirator. Indoor air quality survey monitored for volatile organic compounds, and temperature, carbon dioxide, relative humidity, and carbon monoxide.

After the sanitation process is complete, the trellises are moved to a designated clean room. The respirators are reinspected for structural defects. Damaged respirators are discarded, replaced with new respirators, which are labeled with the workers’ names and employee identification numbers and placed in clear plastic sleeves for redistribution. Moisture content is measured with a moisture meter and results are expressed as percent moisture content (Mini Ligno DX, Lignomat, Portland, OR). The meter is placed directly on the surface of the mask to obtain the reading. Intact treated respirators are marked to indicate the total number of sanitation cycles the respirators have undergone, placed in a sleeve, and loaded into a clean bin for reuse and subsequent redistribution to the workers. The N95 respirators are returned to the assigned unit. The name on the label is used to organize the clean bin by alphabetizing the masks, and the workers reuse only their own previously used masks.

Fit testing can be qualitative or quantitative. Quantitative fit testing provides data and measurements that may be more effective than qualitative fit-test methods that rely on detection of odor or irritation by wearers from introduced molecules (ie, Bitrex, saccharin). The PortaCount Respirator Fit Tester model 8038 (TSI, Incorporated, Shoreview, MN, USA) is a commercially available device that calculates a “fit factor.” Fit factor measures respirator fit during a simulation of workplace activities, including normal breathing, moving the head side to side/up and down, speaking, and bending motion. It is expressed as the challenge aerosol concentration outside the respirator divided by the challenge aerosol concentration that leaks inside the respirator during the test. In addition, to verify maintenance of fit after use, we conducted qualitative fit testing after workers in the sanitation facility had worn a respirator for a shift and the respirator had undergone UV sanitation. Processing cycle time (inspection, loading. sanitation, reinspection, and packing) was measured with a stopwatch to facilitate scalability estimations. Institutional board review approval was not required for this methodological study.

## Results

**Fig. 3.**
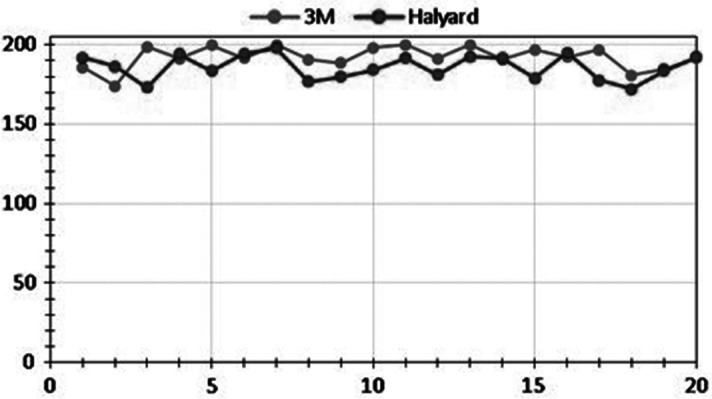
Fit factor by number of cycles. The horizontal axis represents number of cycles. The vertical axis represents fit factor. The tested masks were produced by 3M and Halyard.

The process we report here has demonstrated UV dosing levels that are sufficient to ensure the sanitation of N95 respirators. We measured cumulative doses of >1,000 mJ/cm^2^ UV radiation on the front (patient-facing) sides of the respiratorsand >1000mJ/cm^2^ UV radiation on the back (healthcare worker-facing) sides. Representative quantitative fit-testing results demonstrated no significant degradation of material properties or filtration capacity of the respirators. Quantitative fit testing with OSHA protocol 29CFR1919.134 demonstrated an average fit factor score of 195 after 20 cycles of sanitation (Fig. 3). A passing score is considered to be ≥100. A sample of 12 employees also participated in daily qualitative fit testing with 100% passing after 18 cycles of use and sanitation. Respirator moisture content average was 9.8% (mean), representing significantly more moisture content after use and sanitation compared with the average value for new masks out of the box of 4.8% (*P* < .0001) but far less than the ambient room humidity.

Our total processing cycle time is 38 minutes, enabling the sanitation of an estimated 12,000 respirators per day. We currently have 24 FTEs dedicated to this process. Importantly, a supply of 18 trellises allows for multiple batches in the workflow pipeline, further increasing efficiency. Our process successfully enabled 87.5% of respirators to be returned to their owners within 1 day. To date, 1,230 of 13,049 masks (8%) submitted for sanitation have been discarded due to failure on initial inspection. Respirator failure modes preventing reprocessing included inner mask soilage with makeup (996, 88.9%), physical damage from storage and transport (184, 14.96%), outer mask soilage (43, 3.5%), and strap failure (7, 0.57%).

## Discussion

The shortage of PPE and N95 respirators during the COVID-19 pandemic has forced us to develop novel methods of reuse and sanitation of the N95 respirator.^7,8,12,19^ Because aerosolized droplets are a primary mode of transmission for SARS-CoV-2, high-filtration respirators are an essential means of protecting healthcare workers.^6,12^ The process we developed utilizes UV light and appears to be safe and effective while maintaining respirator filtration efficacy.^15,19^ With repeated UV sanitation cycles and use, we expect the respirators to fail structurally, including the elastic respirator straps or shape of the filter (which will likely affect fit).^18^ Respirator integrity and fit after repeat sanitation and use cycles are quantitatively measured for a variety of respirator models and styles. Masks are discarded if safe performance has been impaired.

This process can be readily implemented; has high throughput; is scalable and reproducible; and demonstrates sanitation while maintaining the filtration performance of the respirators. Furthermore, many hospitals already utilize UV sanitation devices to reduce the transmission of common nosocomial illnesses such as *C. difficile*.

The strengths of our process include simplicity, relatively low cost for implementation, and evidence-based protocol development. Since the effectiveness of UV sanitation is affected by the nature of the surfaces it is used to treat, which are primarily nonporous, a major study limitation is that we did not test masks for SARS-CoV-2 viral growth after sanitation. Viral testing, either by PCR detection or by viral growth in culture, was not performed at the time of UV light respirator sanitation implementation. The presence of SARS-CoV-2 by PCR detection cannot distinguish between live or dead virus and would not discern the effectiveness of UV light sanitation. Viral culture for SARS-CoV-2 is not available in our institution. The dosing we measured has been shown to be effective for the inactivation of this virus.^21^ In addition, multiple tests were performed during development to verify UV dosing and respirator integrity, in addition to the ongoing logging of data for every batch to maintain quality control. Environmental safety measures and strict adherence to protection of personnel were undertaken with diligence.

Barriers to implementation include cultural acceptance of the reuse of disinfected respirators and healthcare worker compliance with proper use guidelines, including the recommendation not to wear makeup, which damages the respirators. Donning and doffing techniques for PPE, with the potential for self-contamination, is another area of concern and may result in decreased acceptance of reusing N95 masks. The authors acknowledge that reuse of “single-use” N95 respirators is certainly not ideal although the current crisis and national shortage situation mandates alternative strategies for healthcare worker protection. The Centers for Disease Control and Prevention (CDC) and the US Food and Drug Administration (FDA) have provided guidance on discretionary reuse of filtering facepiece respirator, noting that reuse is limited by fit, filtration performance, contamination and soiling, and damage.^20^ The method described in this manuscript may be difficult to implement at some centers without the space, equipment, and personnel available to carry out the process, and those centers may elect to utilize other processes for N95 sanitation. UV sanitation of N95 respirators utilizing a quality-controlled, high-throughput process offers a potential means of safe and effective reuse of essential PPE during a national crisis when N95 respirators are in short supply. The N95 respirators passed quantitative and qualitative fit testing through 20 cycles of sanitation and use.

## Supplementary material

For supplementary material accompanying this paper visit https://doi.org/10.1017/ice.2021.37. click here to view supplementary material
