# Supplementary figures and images for: Rationale and process for N95 respirator sanitation and reuse in the coronavirus disease 2019 (COVID-19) pandemic
Source: Infect Control Hosp Epidemiol. 2021 Feb 2:1–5. doi: 10.1017/ice.2021.37 (PMC8712955; doi:10.1017/ice.2021.37)

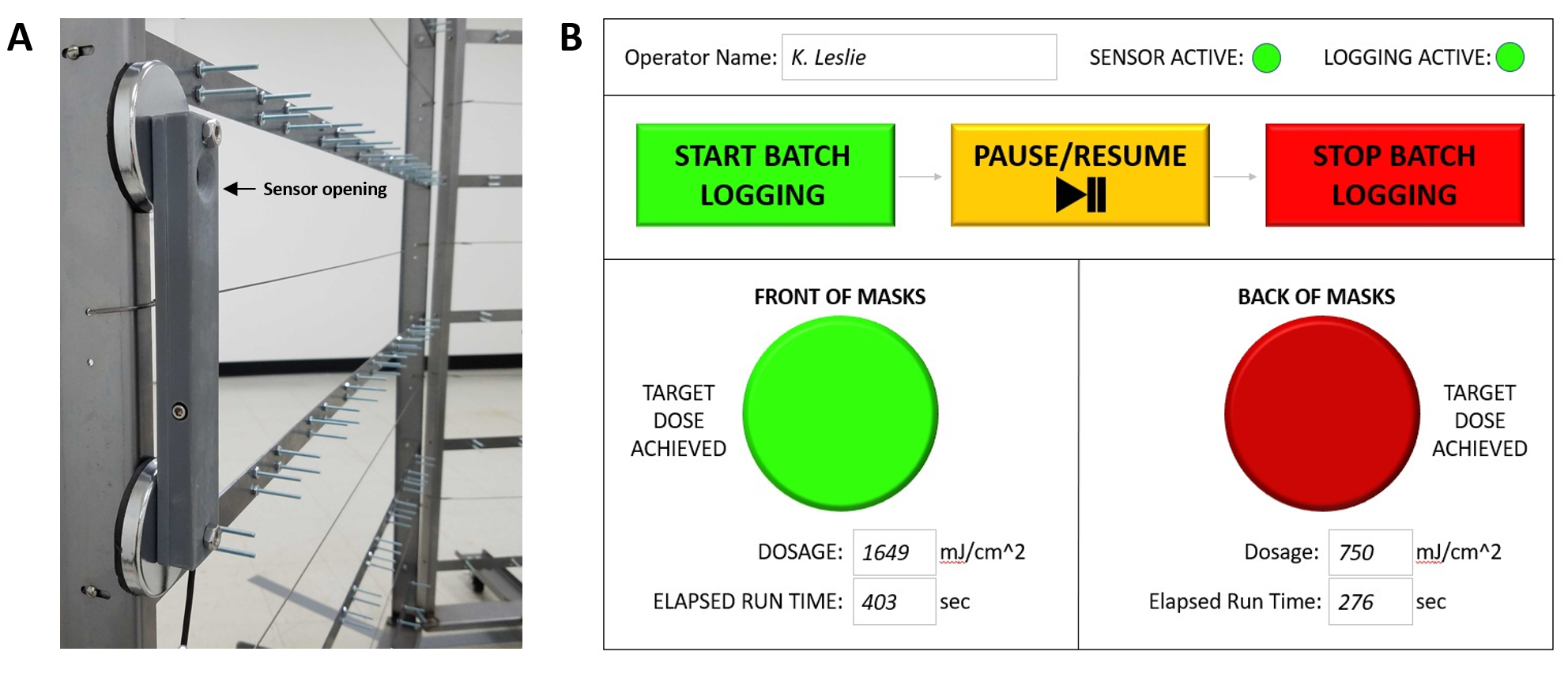

Supplement: Supplementary file 1 [file icesup.zip › S0899823X21000374sup001.tif]
